# Traffic Behavior Recognition Using the Pachinko Allocation Model

**DOI:** 10.3390/s150716040

**Published:** 2015-07-03

**Authors:** Thien Huynh-The, Oresti Banos, Ba-Vui Le, Dinh-Mao Bui, Yongik Yoon, Sungyoung Lee

**Affiliations:** 1 Department of Computer Engineering, Kyung Hee University, Suwon 446-701, Korea; E-Mails: thienht@oslab.khu.ac.kr (T.H.-T.); oresti@oslab.khu.ac.kr (O.B.); lebavui@oslab.khu.ac.kr (B.-V.L.); mao.bui@khu.ac.kr (D.-M.B.); 2 Department of Multimedia Science, Sookmyung's Women University, Seoul 140-742, Korea; E-Mail: yiyoon@sookmyung.ac.kr

**Keywords:** traffic behavior modeling, closed-circuit television (CCTV) system, pachinko allocation model, video-based road surveillance

## Abstract

CCTV-based behavior recognition systems have gained considerable attention in recent years in the transportation surveillance domain for identifying unusual patterns, such as traffic jams, accidents, dangerous driving and other abnormal behaviors. In this paper, a novel approach for traffic behavior modeling is presented for video-based road surveillance. The proposed system combines the pachinko allocation model (PAM) and support vector machine (SVM) for a hierarchical representation and identification of traffic behavior. A background subtraction technique using Gaussian mixture models (GMMs) and an object tracking mechanism based on Kalman filters are utilized to firstly construct the object trajectories. Then, the sparse features comprising the locations and directions of the moving objects are modeled by PAM into traffic topics, namely activities and behaviors. As a key innovation, PAM captures not only the correlation among the activities, but also among the behaviors based on the arbitrary directed acyclic graph (DAG). The SVM classifier is then utilized on top to train and recognize the traffic activity and behavior. The proposed model shows more flexibility and greater expressive power than the commonly-used latent Dirichlet allocation (LDA) approach, leading to a higher recognition accuracy in the behavior classification.

## 1. Introduction

Human behavior analysis (HBA), an integral component of many video surveillance systems, is a research area that has recently attracting attention from the computer vision and artificial intelligence communities. The aim of visual surveillance is to detect, recognize and track moving objects from image sequences and to further understand and describe object behaviors. Visual surveillance in dynamic scenes has been considered in a wide range of potential applications [1], such as security guard services in smart buildings [2–4], traffic surveillance in urban areas [5,6] and access control in specific places [7]. In surveillance applications involving people or vehicles, the behaviors can be analyzed based on the human postures [8–11], the object trajectories [12,13] and the tracking information [14]. This information can be combined to recognize more complex contexts, such as vehicle interactions [15,16], human interactions [17,18] and human to vehicle interactions [19]. Given the large amount of surveillance video data available from closed-circuit television (CCTV) systems and the real-time nature of surveillance applications, it is desirable to provide an automatic operating system that may reduce human intervention as much as possible.

One of the most important applications of surveillance systems, automatic road surveillance, has received increasing interest in recent years. In this domain, the learning of the traffic behavior appears to be the most complex task, especially in highly dynamic environments [5]. A behavior is basically represented through the combination of atomic activities, which are modeled from object features, such as location, direction or tracking information, among others. In order to learn the behavior, the correlation between potential features in the spatial and temporal dimension is normally used as part of probabilistic graphical models [16]. The most widely-used probabilistic approach is the hidden Markov models (HMMs), in which the unknown behaviors are derived from the combination of sequential states with a given likelihood. Although HMM is a simple and efficient model for sequential state estimation, its performance in terms of recognition accuracy tends to degrade in the case of complex scenarios, including multiple objects and long-term temporal behaviors [14]. This limitation has motivated the recent use of topic models as a more effective solution.

Compared with previous works, the authors propose a method for traffic behavior learning for multi-object environments. Firstly, the feature-book, including object trajectories, is created from sparse tracking information in the temporal-spatial dimension. The foreground containing moving objects is extracted using a background subtraction technique based on the Gaussian mixture models (GMM). The Kalman filter is then utilized to track the trajectories of the detected objects in frame sequences. Traffic activities and behaviors are then generated from the identified trajectories with a flexible topic model, namely the pachinko allocation model (PAM). PAM provides a full correlation between features and activity and activities and behavior based on an arbitrary directed acyclic graph (DAG) structure. Finally, a multi-class support vector machine (SVM) technique is employed to classify the activity and behavior according to the outputs of the PAM model. The remainder of this paper is organized as follows. Section 2 provides the discussion of related works. Section 3 describes the proposed method for modeling and identification of traffic behavior. The experimental setup, results and discussion are presented in Section 4. Finally, the conclusions of this paper are summarized in Section 5.

## 2. Related Work

Diverse contributions have been made in the development of behavior recognizers for smart building surveillance applications. The switching hidden semi-Markov model (S-HSMM), an extended version of HMMs, was first introduced by Duong *et al.* [12] for learning and recognizing daily living human activities. The activities are modeled in two stages—presentation and duration—using HSMMs in the bottom layer and the presentation of the sequence of atomic activities in the top layer. To detect abnormal behaviors in indoor environments, a sparse reconstruction analysis of movement trajectories was proposed by Li *et al.* [13]. Although this approach is efficient for small training sets, its performance is sensitive to the numbers of control points used in the cubic B-spline curve approximation, especially for large training sets. Xiang *et al.* [20] proposed a novel framework developed for automatic behavior profiling and online abnormalcy detection using a dynamic Bayesian network (DBN). In this work, the behavior patterns are grouped by using the relevant eigenvectors of the normalized affinity matrix. Unlike some existing techniques, the present approach is apparently simple and robust, even with outliers in the input data. A two-stage learning algorithm based on the time-delayed probabilistic graphical model (TD-PGM) was formulated by Loy *et al.* [21] to effectively detect and localize unusual global events as context-incoherent patterns. Unlike other approaches, the proposed scheme in [22] detects multi-camera group activities from intra- and inter-cameras without a topology inference. The context is characterized by the structure of the hidden variables, which are developed from a discriminative graphical model (DGM). However, it has the drawback of the co-occurrence relationship being captured among activity patterns, which cannot be completely obtained. Rather than propose a novel learning model, Huang *et al.* [23] focused on improving the positioning accuracy by combining the head location and posture recognition as a multi-camera positioning algorithm.

In automatic road surveillance, the vehicle activities and behaviors are detected and recognized for monitoring and warning purposes. A simple method of robustly detecting moving objects was suggested by Kermani *et al.* [6] for recognition of abnormalities in both indoor and outdoor scenarios. The Bayesian change detection (BCD) algorithm is used to eliminate noise, shadows, illumination variations and repeated motions on the background. To produce an analysis of the behavior of moving objects, a generic framework [24] was constructed with two modular blocks: the first is moving region detection and tracking; the second is the integration of the trajectory and geospatial context. A combination of Bayesian computer vision system (BCVS) and coupled hidden Markov models (CHMMs) [14] was suggested for modeling of pedestrian interactions in outdoor environments. In another study, DBN was used for behavior recognition in a road detection system (BRRD) [25] through vehicle sensor networks (VSNs) to infer road events. Moreover, group detection using collaborative filtering provides an improvement in detection performance. HMMs were also applied by Brand *et al.* [15] to organize observed activities based on minimizing the entropy of component distributions for both office activities and outdoor traffic flows. This framework was further adapted to infer the hidden state from ambiguous videos by referencing human body orientations and poses. In [5], Xiang *et al.* recommended a dynamically multi-linked hidden Markov model (DML-HMM) comprising expectation-maximization (EM) clustering and the Bayesian information criterion (BIC) classification. Moreover, dynamic probabilistic networks (DPNs) have been formulated to model the temporal and causal correlations among discrete events for holistic scene-level behavior interpretation. To unify simple and complex action recognition, Sanroma *et al.* [26] encoded simple action HMMs within a stochastic grammar that models complex actions. This approach substantially improved the classification accuracy by developing the higher activity layers based on the recognition of simple actions. Another framework using an extension of stochastic context-free grammar (SCFG) to model the complex temporal relations between atomic activities was suggested by Zhang *et al.* [27]. Their main innovation was a multithread parsing algorithm adopted for the trained event rule induction for recognition instead of the common parser. The common limitation of most of these HMM-based approaches refers to the need of large amounts of training data, since they do not scale well for complex behavior cases.

The use of topic models for context learning has recently been introduced. Zhao *et al.* [16] suggested an effective framework comprised of three steps: construct the motion frame codebook, compose the atomic activities through the LDA-based topic model and classify the behavior with the rough set scheme. Detecting and recognizing urban activity using topic models from online geo-location data was proposed by Hasan *et al.* [28]. Two well-known topic modeling algorithms, the latent Dirichlet allocation (LDA) and hierarchical Dirichlet process (HDP), have been typically employed in HBA systems. The delta-dual hierarchical Dirichlet process (dDHDP), which is an extension of HDP, was designed by Haines *et al.* [29] for jointly learning both normal and abnormal behavior using weakly supervised training examples. A new topic model is introduced by Hospedales *et al.* [30] to overcome the drawbacks on the sensitivity, robustness and efficiency of object behavior mining. The topic model, namely the Markov clustering topic model (MCTM), builds on existing dynamic Bayesian network models and Bayesian topic models. This model was demonstrated to succeed on the unsupervised mining of behaviors in complex and crowded public scenes. Three hierarchical Bayesian models [31]—the LDA mixture model, the HDP mixture model and the dual-HDP model—were proposed in Wang's research. These models cluster both motion pixels and video clips into atomic activities and into interactions. The atomic activities are modeled as distributions over low-level visual features, such as the location and direction of motion pixels, while multi-agent interactions are modeled as distributions over atomic activities. Although many challenging visual surveillance tasks have been completed in the above research, the task of considering activities and interactions with complicated temporal structures remains.

## 3. Methodology

**Figure 1:**
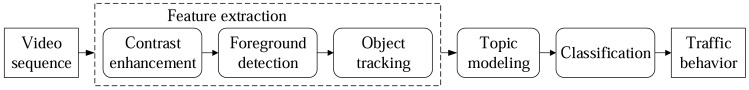
Proposed traffic behavior recognition workflow.

The proposed method consists of the following modules: feature extraction, topic modeling and classification, as presented in Figure 1.

### 3.1. Feature Extraction

As a preprocess for improving the quality of input video sequences, an efficient histogram equalization [32] is used to enhance the overall contrast. The object trajectories in the input video captured from the CCTV system are then extracted using a combined background subtraction and tracking technique. The adaptive-K Gaussian mixture model (AK-GMM) [33] is used to establish the model for background estimation on account of its robustness under changing environments. The moving objects are distinguished from the foreground using a background subtraction technique [34]. As a result, the object coordinates are obtained as the centroid point of the bounding box surrounding the potential object. The Kalman filter is used for tracking objects, and it enables the prediction of an object's future location, a reduction of noise introduced by inaccurate detections and facilitation of the association of multiple objects to their tracks.

**Figure 2:**
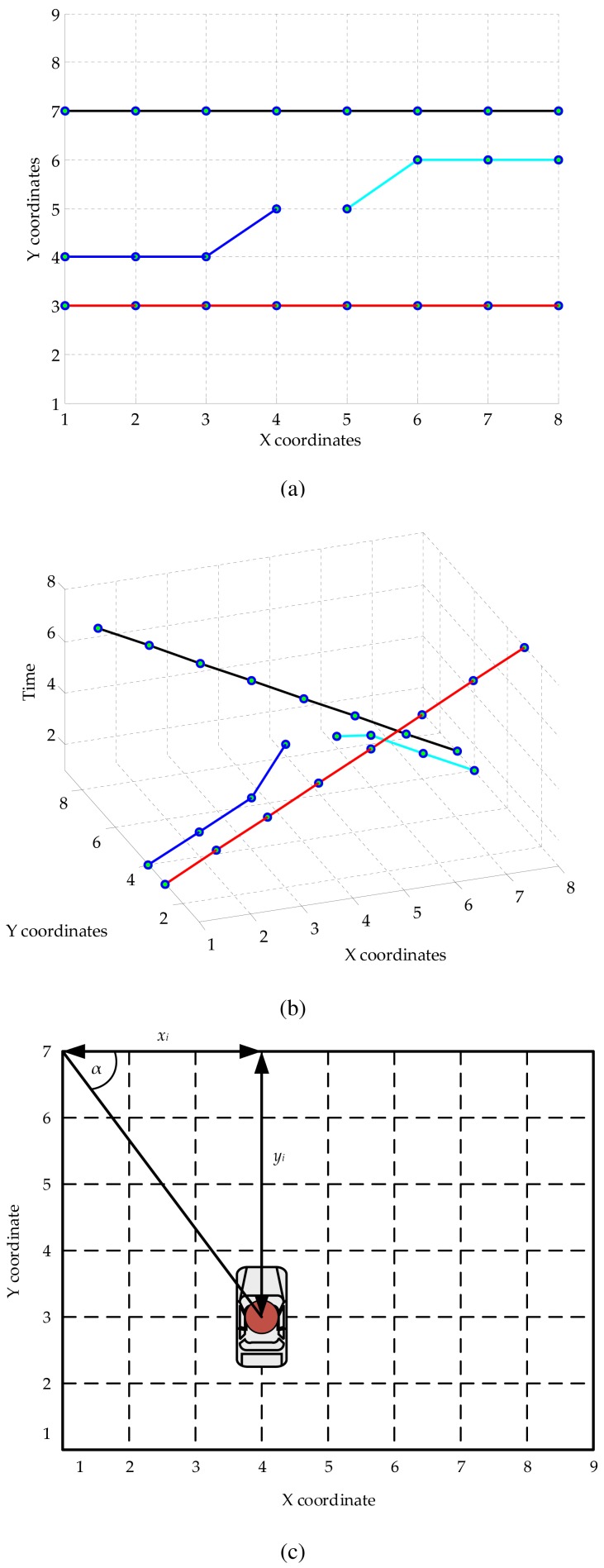
The object trajectory: (**a**) in the spatial dimension (**b**) in the temporal-spatial dimension; and (**c**) the direction of motion path.

The object trajectories are represented in the temporal-spatial dimension. Example object trajectories illustrated in the spatial domain are shown in Figure 2a; those in the temporal-spatial domain are shown in Figure 2b. To determine the orientation of the object trajectory, the absolute angle *α* of the current location is calculated through the following equation:

(1) $$\alpha_{i}=\arcsin\left(\frac{y_{i}}{\sqrt{x_{i}^{2}+y_{i}^{2}}}\right)$$

where (*x_i_*, *y_i_*) are the coordinates of the object at the *i*-th frame. A direction computation example is shown in Figure 2c. Only one angle value corresponding to the current frame is acquired. Each moving object is described by two features: the location and the direction. During a specific time period of the input video, which is presented under the number of input frames from *t_a_* to *t_b_*, the trajectory of an object is formed as:

(2) $$O_{k}^{t_{a-b}}=\left[\left(x_{k}^{t_{a}},y_{k}^{t_{a}},\alpha_{k}^{t_{a}}\right),\left(x_{k}^{t_{a}+1},y_{k}^{t_{a}+1},\alpha_{k}^{t_{a}+1}\right),\ldots ,\left(x_{k}^{t_{b}},y_{k}^{t_{b}},\alpha_{k}^{t_{b}}\right)\right]$$

where $x_{k}^{t_{a}}$ and $y_{k}^{t_{a}}$ are the *X* and *Y* coordinate, respectively. $\alpha_{k}^{t_{a}}$ is the moving direction of the *k*-th detected object at current frame *t_a_*. The object *O_k_* presents the trajectory vector in (*t_b_* − *t_a_*) frames. Assuming that each input video has *n* frames, the trajectory is defined as follows:

(3) $$O_{k}^{n}=\left[\left(x_{k}^{1},y_{k}^{1},\alpha_{k}^{1}\right),\left(x_{k}^{2},y_{k}^{2},\alpha_{k}^{2}\right),\ldots ,\left(x_{k}^{n},y_{k}^{n},\alpha_{k}^{n}\right)\right]$$

The features extracted from the video can be expressed as the feature-book 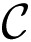:

(4) $$C=\left[\begin{array}{c} \left(x_{1}^{1},y_{1}^{1},\alpha_{1}^{1}\right),\left(x_{1}^{2},y_{1}^{2},\alpha_{1}^{2}\right),\ldots ,\left(x_{1}^{n},y_{1}^{n},\alpha_{1}^{n}\right)\\ \left(x_{2}^{1},y_{2}^{1},\alpha_{2}^{1}\right),\left(x_{2}^{2},y_{2}^{2},\alpha_{2}^{2}\right),\ldots ,\left(x_{2}^{n},y_{2}^{n},\alpha_{2}^{n}\right)\\ \vdots \\ \left(x_{K}^{1},y_{K}^{1},\alpha_{K}^{1}\right),\left(x_{K}^{2},y_{K}^{2},\alpha_{K}^{2}\right),\ldots ,\left(x_{K}^{n},y_{K}^{n},\alpha_{K}^{n}\right) \end{array}\right]=\left[\begin{array}{c} O_{1}^{n}\\ O_{2}^{n}\\ \vdots \\ O_{K}^{n} \end{array}\right]$$

where *K* is the number of detected objects.

### 3.2. Topic Modeling

In this work, the traffic behavior is defined as the collection of activities in which an object trajectory is automatically assigned into an activity class. A short video can contain several trajectories that can be classified into the same activity class; *i.e.*, they correlate in terms of location and/or direction. Therefore, it is important to model object trajectories in the correlative activities and to automatically model activities in the satisfactory behavior.

The pachinko allocation model (PAM) [35] is a hierarchical generative model considered here to define behaviors from the combination of features from moving objects. PAM was firstly suggested for use in the machine learning and natural language processing as a topic model. In its original application, PAM models correlations between topics in addition to word correlations and, thereby, establishes topics. To represent and learn arbitrary, nested and possibly sparse topic correlations, this model utilizes an arbitrary directed acyclic graph (DAGs) structure. Furthermore, compared to LDA [36], PAM provides more flexibility and greater expressive power than LDA, since it captures not only the correlations among the words, like in LDA, but also the correlations among topics.

**Figure 3:**
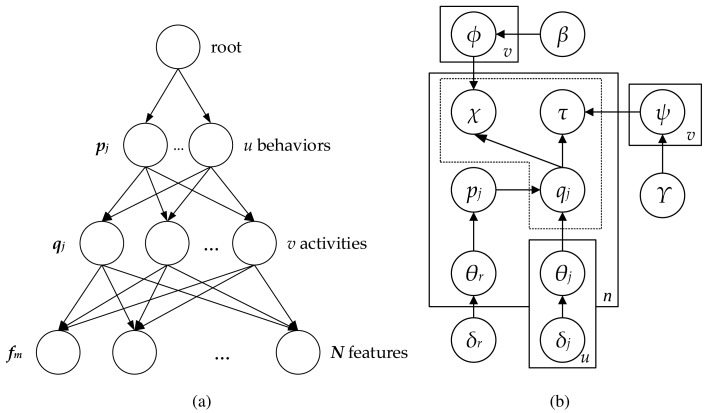
Pachinko allocation model: (**a**) hierarchical topic model (**b**) graphic model.

In the following subsection, the details of the proposed model based on PAM are introduced with the algorithm for the estimation of the parameters. Although PAM employs arbitrary DAGs to model the topic correlations, this work proposes a four-level hierarchy structure as a special case of PAM [37]. This structure consists of one root topic, *u* super topics at the second level 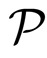 = {*p*_1_, *p*_2_, …, *p_u_*}, *v* subtopics at the third level 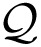 = {*q*_1_, *q*_2_, …, *q_v_*} and the words at the bottom. Words refer here to the object features comprising the location and direction information, which were organized in the previous stage. The super topic and subtopic correspond to the traffic behavior and activity, respectively. The root is associated with behaviors; the behaviors are fully associated with activities; and the activities are fully connected to the features, as shown in Figure 3a. The multinomials of the root and behaviors are sampled for each frame based on a single Dirichlet distribution *g_r_* (*δ_r_*) and ${q_{j}\left(\delta_{j}\right)|}_{j=1}^{u}$, respectively. The activities are modeled with multinomial distributions ${\phi_{q_{j}}|}_{j=1}^{v}$ and ${\psi_{q_{j}}|}_{j=1}^{v}$ sampled from Dirichlet distribution *g* (*β*) and *g* (*γ*), which are used for sampling the location and direction features. Figure 3b depicts a graphic model for the four-levels PAM. The particular notations used in PAM are summarized in Table 1. According to the standard PAM [35], considered a scene as a document *d* consisting of a the sequence of *n* frames 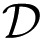 = {*d*_1_, *d*_2_, …, *d_n_*}, this is modeled as follows:

1. Sample a multinomial distribution $\theta_{r}^{\left(d\right)}$ from a Dirichlet prior $\delta_{r}^{\left(d\right)}$ for each scene *d*.
2. For each behavior *p_j_*, sample a multinomial distribution $\theta_{p_{j}}^{\left(d\right)}$ from *g_j_* (*δ_j_*) in which $\theta_{p_{j}}^{\left(d\right)}$ is a multinomial distribution over activities.
3. Sample multinomial distributions ${\phi_{q_{j}}|}_{j=1}^{v}$ from a Dirichlet prior *β* for each activity *q_j_*.
4. Sample multinomial distributions ${\psi_{q_{j}}|}_{j=1}^{v}$ from a Dirichlet prior *γ* for each activity *q_j_*.
5. For the *m*-th feature in the current scene *d* of the object *O_k_*:
  - Sample a behavior *p_m,d,O_k__* from $\theta_{r}^{\left(d\right)}$ and an activity *q_m,d,O_k__* from $\theta_{p_{m,d,O_{k}}}^{\left(d\right)}$
  - Sample a location feature *χ_m,d,O_k__* from multinomial *ϕ_q_m,d,O_k___* and a direction feature *τ_m,d,O_k__* from multinomial *ψ_q_m,d,O_k___*.

Following this process, the joint probability of the generated scene *d*, the behavior assignments *p*^(^*^d^*^)^, the activity assignments *q*^(^*^d^*^)^ and the multinomial distribution *θ*^(^*^d^*^)^ is calculated as:

(5) $$P\left(d,q^{\left(d\right)},p^{\left(d\right)},\theta^{\left(d\right)}|\delta ,\beta ,\gamma \right)=P\left(\theta_{r}|\delta_{r}\right)\prod_{j=1}^{u}P\left(\theta_{p_{j}}^{\left(d\right)}|\delta_{j}\right)\prod_{m}\left(P\left(p_{m}|\theta_{r}^{\left(d\right)}\right)P\left(q_{m}|\theta_{p_{m}}^{\left(d\right)}\right)P\left(f_{m}|\phi_{q},\psi_{q}\right)\right)$$

where *P* (*f_m_*|*ϕ_q_*, *ψ_q_*) = *P* (*χ_m_*|*ϕ_q_*) *P* (*τ_m_*|*ψ_q_*). Integrating out *θ*^(^*^d^*^)^ and summing over *p*^(^*^d^*^)^ and *q*^(^*^d^*^)^ the marginal probability of each scene can be calculated as:

(6) $$P\left(d|\delta ,\beta ,\gamma \right)=\int P\left(\theta_{r}^{\left(d\right)}|\delta_{r}\right)\prod_{j=1}^{u}P\left(\theta_{p_{j}}^{\left(d\right)}|\delta_{j}\right)\prod_{m}\underset{p_{m},q_{m}}{\text{∑}}\left(P\left(p_{m}|\theta_{r}^{\left(d\right)}\right)P\left(q_{m}|\theta_{p_{m}}^{\left(d\right)}\right)P\left(f_{m}|\phi_{q},\psi_{q}\right)\right)d\theta^{\left(d\right)}$$

The probability of generating the corpus 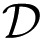 is computed by:

(7) $$P\left(D|\delta ,\beta ,\gamma \right)=\int \prod_{j=1}^{v}\left(P\left(\phi_{q_{j}}|\beta \right)+P\left(\psi_{g_{j}}|\gamma \right)\right)\prod_{d}P\left(d|\delta ,\beta ,\gamma \right)d\phi d\psi$$

The approximate inference result of the condition distribution that samples the behavior and activity assignments for each feature can be obtained as:

(8) $$\begin{array}{ll} P\left(p_{m},q_{m}|D,P_{-m},Q_{-m},\delta ,\beta ,\gamma \right) & \propto P\left(m,p_{m},q_{m}|D_{-m},P_{-m},Q_{-m},\delta ,\beta ,\gamma \right)\\ & =\frac{P\left(D,P,Q|\delta ,\beta ,\gamma \right)}{P\left(D,P_{-m},Q_{-m}|\delta ,\beta ,\gamma \right)}\\ & =\frac{n_{j}^{\left(d\right)}+\delta_{rj}}{n_{r}^{\left(d\right)}+\overset{u}{\underset{j=1}{\text{∑}}}\delta_{rj}n_{j}^{\left(d\right)}}\times \frac{n_{jl}^{\left(d\right)}+\delta_{jl}}{n_{j}^{\left(d\right)}+\overset{v}{\underset{l=1}{\text{∑}}}\delta_{jl}}\times \frac{n_{lh}+\beta_{h}}{n_{l}+\overset{N}{\underset{h=1}{\text{∑}}}\beta_{h}}\times \frac{n_{lz}+\gamma_{z}}{n_{l}+\overset{M}{\underset{z=1}{\text{∑}}}\gamma_{z}} \end{array}$$

Hyper-parameters *δ*, *β* and *γ* can be estimated via the Gibbs sampling algorithm, which is described in [35]. As in [35], the notation −*m* denotes behavior assignments, except for the *m*-th feature. After modeling, the new data obtained by tagging the motion location and direction are generated. By merging the same feature items for different video contents, the probability distribution is obtained as an implicit activity-behavior-frame sequence matrix. The posterior is maximized by multiplying the direction probability of all locations from their corresponding subtopic location distributions.

### 3.3. Classification

**Figure 4:**
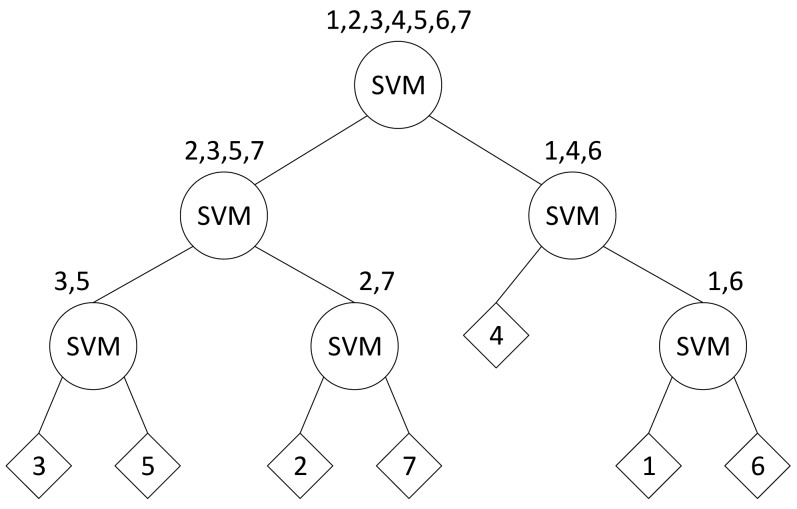
Illustration of SVM-binary tree architecture (BTA).

Based on the PAM-based topic modeling, every video sequence can be represented through a *u* × *v* matrix, where *u* is the number of behaviors and *v* is the number of activities. To train the classifier, the labels of vectors and matrices are manually denoted with their classes manually In this paper, the authors use a SVM with binary tree architecture (SVM-BTA) [38] to solve the *N*-class pattern recognition problem. An illustration of SVM-BTA is shown in Figure 4. Each node in the architecture makes a binary decision using the original SVM. By recursively dividing the classes into two disjointed groups in each node of the decision tree, the SVM classifier decides the group to which the unknown samples that should be assigned. The class is determined by a clustering algorithm according to the class membership and the inter-class distance. Although *N* − 1 SVMs are trained for an *N*-class problem, only log_2_*N* SVMs are consulted at most to classify a sample. This approach requires fewer binary SVMs than popular methods, such as *N* (*N* − 1)/2 SVMs in the one-against-one approach and *N* SVMs in the one-against-others approach. Moreover, both approaches have the drawback of very expensive computational cost requirements and accuracy degradation [38]. An essential contribution of the SVM-BTA approach, the multiclass issue, is converted into binary-tree architectures without performance reduction. Moreover, a dramatic improvement in recognition speed can be achieved for increasing the number of classes.

## 4. Experimental Evaluation

### 4.1. Experimental Setup

**Figure 5:**
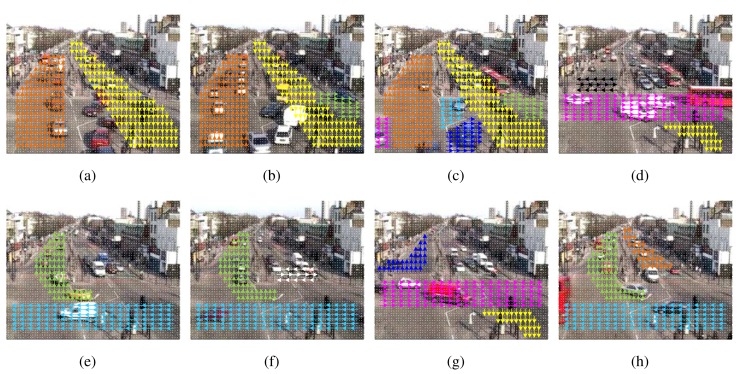
Traffic activities discovered by PAM. (**a–c**) The vertical traffic behavior; (**d–h**) the horizontal traffic behavior.

The experiments were performed on the QMUL (Queen Mary University of London) dataset [39], which includes a long-term video recorded at 25 fps for the frame rate and 360 × 288 for the frame resolution. Placed at an intersection, the video captured a busy traffic scenario involving a vehicle and pedestrian with dynamic movements. The video sequence was divided into short non-overlapping clips, each of which was 4 s. This duration is more convenient for observing when compared with too long a duration in Hospedales's work [40] (12 s) or two short a duration in Zhao's work [16] (2 s). The length of each clip was set to ensure that a behavior was not covered by others. A total of 750 clips comprised 320 vertical traffic flow clips; 430 horizontal traffic flow clips were tested with the manual activity and behavior labeling. Some activities cannot be fully categorized into horizontal or vertical traffic behavior, for example 40 frames may represent vertical traffic and 60 frames horizontal traffic. For example, a car can move in the vertical traffic from the top, and it will turn left or turn right at the intersection. Therefore, the authors categorized a given clip into either vertical or horizontal behavior based on the duration of the observed behaviors. If both behaviors are present during the whole clip, this is categorized into the most fluent behavior, *i.e.*, with less changes or interruptions. In the vertical traffic, activities were discovered by PAM, as shown in Figure 5a–c. The horizontal traffic activities are presented in Figure 5d–h. Although PAM automatically discovered and modeled sparse words into super topics and subtopics, the number of topics had to be initially set. In this work, *u* = 2 for vertical and horizontal traffic behaviors; and *v* = 14 for traffic activities involving six vertical and eight horizontal activities. The description of the discovered activities outlined in Figure 5 is referenced in Table 2. In the PAM modeling, the Dirichlet distribution over behaviors and activities was produced with the parameter 0.01; the Gibbs sampling was processed with 1000 burn-in iterations. In the SVM-BTA classifier, the Gaussian kernel was used to set up for each node of binary classification. For each vertical and horizontal traffic dataset, the proposed method was evaluated using the 10-fold cross-validation. In order to analyze accuracy of the proposed method, Recall and Precision are used with the confusion matrix of each experiment. All of the experiments were performed on a desktop PC operating Windows 7 with a 2.67-GHz Intel Core i5 CPU and 4 GB of RAM. MATLAB R2013a was the software used for simulation.

### 4.2. Results and Discussion

**Figure 6:**
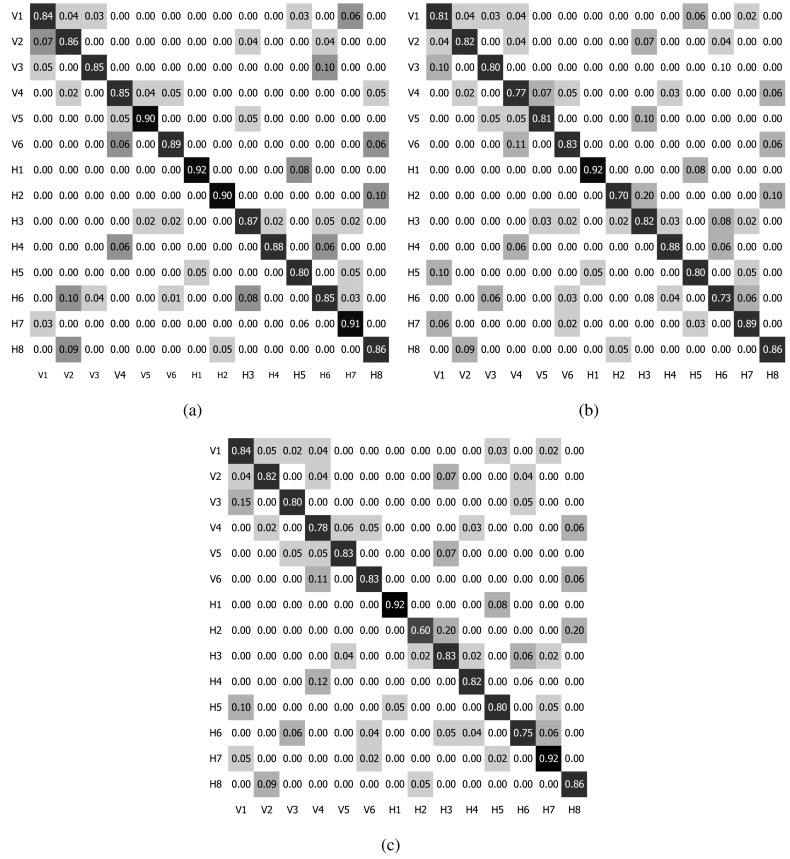
Confusion matrix of the SVM classifier for the mixing of all vertical and horizontal traffic with overall classification accuracy: (**a**) PAM 86.4%; (**b**) LDA 80.4%; and (**c**) MCTM 81.6%.

In the experiments, the authors evaluated the performance in the classification accuracy of the proposed method for the detection of the vertical and horizontal traffic. Moreover, the method was compared with similar approaches using standard latent Dirichlet allocation (LDA) [36] and Markov clustering topic mode (MCTM) [30] for topic modeling. At first, the activity classification was applied to each separate dataset of the vertical and horizontal clips. The confusion matrices of the SVM-BTA classifier using PAM and LDA are reported in Tables 3–5 for the vertical and in Tables 6–8 for the horizontal traffic dataset. The mixture of all vertical and horizontal traffic activity classification results are presented in the confusion matrix shown in Figure 6 with 14 classes in total. Secondly, the behavior classification was evaluated for all clips to identify the category of the input clip. For behavior classification, all clips in the merged dataset were evaluated using the binary SVM classifier. The quantitative results of the evaluated metrics are represented in Table 9. It is important to note that only the binary SVM classifier was utilized for the behavior classification (either vertical or horizontal) instead of the multi-class SVM classifier for the activity case.

In the vertical and horizontal traffic datasets, the numbers of clips presenting particular activities discovered by PAM were not equivalent. For example, the occurrence of activity V1 and V4 in the vertical dataset corresponding to the top-bottom flows consumed more than 66% of the full video length. Similarly, activities H3 and H6 in the horizontal dataset corresponding to left-right flows consumed more than approximately 62% of the video length. Therefore, they can be regarded as the primary activities corresponding to each dataset. Based on the results in the Table 3, activities V3 and V6 obtained the highest accuracies (greater than 94%), because they contained specific features in the given direction. On the other hand, activities V4 and V5 had the worst classification rates, since they easily overlapped by covering similar location and direction features. Six clips were recognized as V5, and five clips were recognized as V6 instead of the correct class of V4. It was evident that the primary activities with high appearance frequencies had larger interesting regions compared with the others. Some small regions with fewer appearance activities were covered by them, which resulted in the classification confusion. This phenomenon likewise occurred in the horizontal dataset with the worst classification results of the primary activities, particularly activities H3 and H6 in Table 5.

In the activity classification using the respective vertical and horizontal traffic clips separately, the proposed method using PAM for topic modeling outperformed the LDA-based method in most tested activities (above 90% of overall accuracy rate). Compared with LDA, PAM provided the higher accuracy rate through the Recall and Precision metrics, especially with the overall Accuracy (greater than 6% in the vertical dataset and 4% in the horizontal dataset). Although obtaining a high classification performance, MCTM overall accuracy degraded along with the increase of the number of classes (90.94% for six vertical classes compared with 88.14% for eight horizontal classes). When merging the vertical and horizontal dataset for activity classification, the accuracy tended to reduce in all evaluated models. From the results in Figure 6, PAM outperformed LDA and MCTM with 86.4% *vs.* 80.4% and 81.6% in terms of Accuracy. When the number of classes was increased in the merging dataset, a strong degradation was again observed in the MCTM model, because it is difficult to correctly classify activities of two or more phases, for example activity V5 including one part of a common activity (top to bottom flow) and another part of unusual activity (turning left at the intersection). MCTM got only 60% in terms of accuracy with rare activity H2, while some common activities are confused with others, such as activity V4 with V5 and H8 and activity H6 with V3 and H7. In the merging of all clips to classify the behavior, MCTM still showed the highest accuracy rate. Concretely, only 76 clips (≈10.1%) were incorrectly recognized by MCTM instead of 93 and 127 clips (≈12.4% and 16.9%) respectively misclassified by PAM and LDA. Despite using the DAGs structure and exploiting the Dirichlet distribution, LDA only captured the correlation among the features to support the high level information (activities or behaviors), because it was constructed by a three-layer model comprising the feature, activity (or behavior) and root layers. Therefore, LDA has difficulty modeling data in which some behaviors co-occurred more frequently than in others. Compared with PAM capturing only spatial correlation between activities, MCTM further measured temporal correlation between events to improve the binary behavior classification. Moreover, the distinction between only two vertical and horizontal behaviors is more explicit under the spatio-temporal dimension. However, MCTM sometimes had a negative effect for allowing a rare activity to occur alongside numerous common activities [30]. This led to the degradation of the classification accuracy in the MCTM model whenever the number of behaviors or activities was increased.

### 4.3. Complexity and Computational Time

It is difficult to provide theoretical analysis for the convergence of Gibbs sampling. Concretely, the time complexity of each Gibbs sampling iteration for LDA was 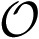 (*Nu*) + 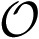 (*Nv*) when modeling *N* features into *u* behaviors and *v* activities. Running on our system, it took less than 2 h to process 750 4-s clips from the UMUL dataset. The Gibbs sampling for PAM was much slower at approximately 3.5 h, because it depended not only on the number of super topics, but also on the number of sub-topics with 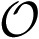 (*Nuv*). Since two layers, including action and behavior, were considered in the MCTM model, it required 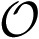 (*u*^2^) + 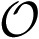 (*Nuv*) time per parameter sample [30]. The total process time of MCTM was quite equivalent to PAM's time owing to the insignificance of the component 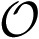 (*u*^2^) in comparison to 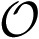 (*Nuv*) of the proposed method.

## 5. Conclusions

In this paper, the authors proposed a behavior recognition method based on a four-level hierarchy PAM model for traffic video surveillance purposes. Two types of features extracted from a traffic video, comprising the location and direction of the moving object, are used to construct the object trajectory. For topic modeling, the PAM algorithm is then used to reorganize the sparse features. The probability distribution, the new data generated from PAM, is then provided for the SVM-BTA classifier. With the advantage of capturing correlations among features, as well as among activities and behaviors, PAM provides more expressive power to support complicated structures, while adopting more realistic assumptions. This property helps improve the classification rate in behavior recognition. In our experimental evaluation, the proposed method is compared with LDA and MCTM in individual datasets of vertical and horizontal traffic, as well as a merged dataset, including both activities and behaviors. PAM outperformed LDA in most of the tests with an accuracy of 90.63% *vs.* 84.06% in the vertical traffic dataset, 90.00% *vs.* 85.58% in the horizontal traffic dataset and 87.60% *vs.* 83.07% in the merged dataset. Although MCTM provided the best results in binary behavior classification, this model showed the limitation of the multiclass problem, especial with complex activities comprising two or more phases. Contrary to MCTM, PAM is preferable in the recognition of rare and complex activities due to the captured correlations among the visual words and topics. For future work, the feature extraction algorithm will be considered to increase the processing speed through complexity reduction.

## Figures and Tables

**Table 1:** Notations used in the pachinko allocation model (PAM) model.

**Symbol**	**Description**
*u*	Number of behaviors
*v*	Number of activities
*n*	Number of frames
*N*	Number of unique locations
*M*	Number of unique directions
*g_r_* (*δ_r_*)	Dirichlet distribution associated with the root
*g_j_* (*δ_j_*)	Dirichlet distribution associated with the *j*-th behavior, *u*-dimensional vector *g_j_*
*g*(*β*)	Dirichlet distribution associated with activity for location feature
*g*(*γ*)	Dirichlet distribution associated with activity for direction feature
$\theta_{r}^{\left(d\right)}$	Multinomial distribution sampled from *g_r_* (*δ_r_*) for the root to scene *d, n*-dimensional vector *θ_r_*
$\theta_{p_{j}}^{\left(d\right)}$	Multinomial distribution sampled from *g_j_* (*δ_j_*) for a behavior to scene *d*, *u* × *n* matrix *θ_p_*
*ϕ_q_*	Multinomial distribution sampled from *g* (*β*) for an activity to scene *d*, *v* × *n* matrix *φ*
*ψ_q_*	Multinomial distribution sampled from *g* (*γ*) for an activity to scene *d*, *v* × *n* matrix *ψ*
*χ_m,d,O_k__*	*m*-th location in the frame *d* of an object *O_k_*, *N × n × k* matrix *χ*
*τ_m,d,O_k__*	*m*-th direction in the frame *d* of an object *O_k_, M* × *n* × *k* matrix τ
*p_m,d,O_k__*	Behavior *p* associated with the *m*-th feature in *d* of *O_k_*, *u*-dimensional vector 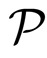
*q_m,d,O_k__*	Activity *q* associated with the *m*-th feature in *d* of *O_k_*, *v*-dimensional vector 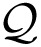
$n_{r}^{\left(d\right)}$	Number of occurrences of the root *r* in the scene *d*
$n_{j}^{\left(d\right)}$	Number of occurrences of the behavior *j* in the scene *d*
*n_l_*	Total number of occurrences of activity *q_l_* in the whole corpus 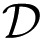
$n_{jl}^{\left(d\right)}$	Number of times that activity *q_l_* is sampled from the behavior *p_j_* in the scene *d*
*n_lh_*	Number of occurrences of location feature *χ* in the activity *q_l_*
*n_lz_*	Number of occurrences of direction feature *τ_z_* in the activity *q_l_*

**Table 2:** Activity descriptions of two main behaviors.

**Vertical Traffic**			
**Activity**	**Color**	Figure 5	**Description**
V1	Orange	(a), (b), (c)	Bottom to top flow
V2	Blue	(c)	Bottom to top and turn left at the intersection
V3	Pink	(c)	Bottom to top and turn right at the intersection
V4	Yellow	(a), (b), (c)	Top to bottom flow
V5	Green	(b), (c)	Top to bottom and turn left at the intersection
V6	Cyan	(c)	Top to bottom and turn right at the intersection

**Horizontal Traffic**			

**Activity**	**Color**	Figure 5	**Description**

H1	Black	(d)	Vertical flow for pedestrian on the left side
H2	White	(f)	Vertical flow for pedestrian on the right side
H3	Pink	(d), (g)	Left to right flow
H4	Yellow	(d), (g)	Left to right and turn right at the intersection
H5	Blue	(g)	Left to right and turn left at the intersection
H6	Cyan	(e), (f), (h)	Right to left flow
H7	Green	(e), (f), (h)	Right to left and turn right at the intersection
H8	Orange	(h)	Top to bottom and stop at the intersection

**Table 3:** Confusion matrix of the SVM classifier using PAM for the vertical traffic.

**Activities**	**V1**	**V2**	**V3**	**V4**	**V5**	**V6**	**Recall (%)**
V1	93	5	3	0	0	0	91.18
V2	2	26	0	0	0	0	92.86
V3	1	0	19	0	0	0	95.00
V4	0	2	0	97	6	5	88.18
V5	0	0	0	4	38	0	90.48
V6	0	0	0	1	0	17	94.44

Precision (%)	96.88	78.79	86.36	95.10	86.36	73.91	

Accuracy (%)	**90.63**						

**Table 4:** Confusion matrix of the SVM classifier using LDA for the vertical traffic.

**Activities**	**V1**	**V2**	**V3**	**V4**	**V5**	**V6**	**Recall (%)**
V1	89	4	1	7	0	1	87.25
V2	0	25	0	3	0	0	89.29
V3	3	0	17	0	0	0	85.00
V4	9	2	0	88	8	3	80.00
V5	0	0	3	4	35	0	83.33
V6	0	0	0	3	0	15	83.33

Precision (%)	88.12	80.65	80.95	83.81	81.40	78.95	

Accuracy (%)	**84.06**						

**Table 5:** Confusion matrix of the SVM classifier using Markov clustering topic model (MCTM) for the vertical traffic.

**Activities**	**V1**	**V2**	**V3**	**V4**	**V5**	**V6**	**Recall (%)**
V1	92	4	2	3	0	1	90.20
V2	1	27	0	0	0	0	96.43
V3	5	0	15	0	0	0	75.00
V4	5	0	0	105	2	2	95.45
V5	0	0	3	3	36	0	85.71
V6	0	0	1	1	0	16	88.89

Precision (%)	92.93	87.10	71.43	93.75	94.74	84.21	

Accuracy (%)	**90.94**						

**Table 6:** Confusion matrix of the SVM classifier using PAM for the horizontal traffic.

**Activities**	**H1**	**H2**	**H3**	**H4**	**H5**	**H6**	**H7**	**H8**	**Recall (%)**
H1	11	0	0	0	1	0	0	0	91.67
H2	0	9	0	0	0	0	0	1	90.00
H3	0	0	112	2	0	9	3	0	88.89
H4	0	0	0	31	0	3	0	0	91.18
H5	1	0	0	0	18	0	1	0	90.00
H6	0	0	12	0	0	125	5	0	88.03
H7	0	0	0	0	4	0	60	0	93.75
H8	0	1	0	0	0	0	0	21	95.45

Precision (%)	91.67	90.00	90.32	93.94	78.26	91.24	86.96	95.45	

Accuracy (%)	**90.00**								

**Table 7:** Confusion matrix of the SVM classifier using LDA for the horizontal traffic.

**Activities**	**H1**	**H2**	**H3**	**H4**	**H5**	**H6**	**H7**	**H8**	**Recall (%)**
H1	11	0	0	0	1	0	0	0	91.67
H2	0	9	0	0	0	0	0	1	90.00
H3	1	1	106	4	0	11	3	0	84.13
H4	0	0	0	28	0	6	0	0	82.35
H5	1	0	1	0	17	0	1	0	85.00
H6	0	0	10	5	0	119	8	0	83.80
H7	0	0	0	0	7	0	57	0	89.06
H8	0	1	0	0	0	0	0	21	95.45

Precision (%)	84.62	81.82	90.60	75.68	68.00	87.50	82.61	95.45	

Accuracy (%)	**85.58**								

**Table 8:** Confusion matrix of the SVM classifier using MCTM for the horizontal traffic.

**Activities**	**H1**	**H2**	**H3**	**H4**	**H5**	**H6**	**H7**	**H8**	**Recall (%)**
H1	10	0	1	0	1	0	0	0	83.33
H2	0	9	0	0	0	0	0	1	90.00
H3	1	1	111	4	0	6	3	0	88.10
H4	0	0	2	26	0	6	0	0	76.47
H5	1	0	1	0	17	0	1	0	85.00
H6	0	0	5	3	3	128	3	0	90.14
H7	0	0	0	0	7	0	57	0	89.06
H8	0	1	0	0	0	0	0	21	95.45

Precision (%)	83.33	81.82	92.50	78.79	60.70	91.43	89.06	95.45	

Accuracy (%)	**88.14**								

**Table 9:** Behavior classification comparison between PAM and LDA.

**Behavior**	**PAM**			**LDA** [36]			**MCTM** [30]		
		
	**Vertical**	**Horizontal**	**Recall** (%)	**Vertical**	**Horizontal**	**Recall** (%)	**Vertical**	**Horizontal**	**Recall** (%)
Vertical	286	34	89.38	259	61	80.94	291	29	90.94
Horizontal	59	371	86.28	66	364	84.65	47	383	89.07

Precision (%)	82.90	91.60	**-**	79.69	85.65	**-**	86.09	92.96	**-**

Accuracy (%)	**87.60**			**83.07**			**89.87**		
